# Preparation of Polyurethane Adhesives from Crude and Purified Liquefied Wood Sawdust

**DOI:** 10.3390/polym13193267

**Published:** 2021-09-25

**Authors:** Wen Jiang, Reza Hosseinpourpia, Vladimirs Biziks, Sheikh Ali Ahmed, Holger Militz, Stergios Adamopoulos

**Affiliations:** 1 Department of Forestry and Wood Technology, Linnaeus University, Lückligs Plats 1, 35195 Växjö, Sweden; wen.jiang@lnu.se (W.J.); reza.hosseinpourpia@lnu.se (R.H.); sheikh.ahmed@lnu.se (S.A.A.); 2 Institute of Wood Biology and Wood Products, Georg-August University Göettingen, Büsgenweg 4, 37077 Göttingen, Germany; vbiziks@gwdg.de (V.B.); holger.militz@uni-goettingen.de (H.M.); 3 Department of Forest Biomaterials and Technology, Swedish University of Agricultural Sciences, Vallvägen 9C, 75007 Uppsala, Sweden

**Keywords:** adhesive penetration, bio-polyol, bond strength, ethylene glycol, FTIR, liquefaction, pMDI, TGA

## Abstract

Polyurethane (PU) adhesives were prepared with bio-polyols obtained via acid-catalyzed polyhydric alcohol liquefaction of wood sawdust and polymeric diphenylmethane diisocyanate (pMDI). Two polyols, i.e., crude and purified liquefied wood (CLW and PLW), were obtained from the liquefaction process with a high yield of 99.7%. PU adhesives, namely CLWPU and PLWPU, were then prepared by reaction of CLW or PLW with pMDI at various isocyanate to hydroxyl group (NCO:OH) molar ratios of 0.5:1, 1:1, 1.5:1, and 2:1. The chemical structure and thermal behavior of the bio-polyols and the cured PU adhesives were analyzed by Fourier transform infrared spectroscopy (FTIR) and thermogravimetric analysis (TGA). Performance of the adhesives was evaluated by single-lap joint shear tests according to EN 302-1:2003, and by adhesive penetration. The highest shear strength was found at the NCO:OH molar ratio of 1.5:1 as 4.82 ± 1.01 N/mm^2^ and 4.80 ± 0.49 N/mm^2^ for CLWPU and PLWPU, respectively. The chemical structure and thermal properties of the cured CLWPU and PLWPU adhesives were considerably influenced by the NCO:OH molar ratio.

## 1. Introduction

The chemistry of polyurethane (PU) was introduced by Otto Bayer and his co-workers by combining polyester polyols with di- and poly-isocyanates in the 1930s [1]. Since then, PU adhesives have undergone a rapid development and they are one of the most important synthetic resins used on the market today. It is either a thermoset or a thermoplastic polymer prepared by the reaction of isocyanates with diols or polyols in the presence of a catalyst, a chain extender, or other additives [2]. PU adhesives have been widely applied to bond different substrates, such as wood, glass, plastic, and ceramics, due to their good wetability on the surfaces of substrates, strong adhesion strength, and good chemical resistance [1,3]. These adhesives are generally prepared from petroleum-based and non-biodegradable synthetic prepolymers [4]. With increasing concerns about global climate change and shortage of fossil fuel resources, polymers derived from renewable resources are ideal sustainable alternatives for providing green polymeric materials, which can degrade after their service life [5]. Over the last two decades, attempts have been made to develop PU adhesives from natural resources, e.g., vegetable oils, lignin, starch, and polyols from the liquefaction process of lignocellulosic materials [6,7,8,9]. Table 1 presents the chronological evolution of research on the development of bio-based polyurethane adhesives including the development of synthesis technology and uses of natural resources.

Liquefaction of biomass in the presence of polyhydric alcohols and acid catalysts is an effective thermochemical conversion method to turn solid biomass into liquids for various applications [40]. A large variety of virgin and waste biomass, such as wood, bark, cork, bagasse, and agriculture crop residues, have been used in liquefaction [40,41]. The main chemical components of biomass are cellulose, hemicelluloses, and lignin, which all contain two or more hydroxyl groups per molecule [5]. Under an acid-catalyzed aqueous environment coupled by heating, hemicelluloses and amorphous cellulose are easily degraded and hydrolyzed, whereas lignin and crystalline cellulose are degraded and decomposed initially to smaller fragments, and further react with themselves or with the solvent to form higher molecular fragments [40,41,42]. These, together with unreacted solvents in the liquefaction products, provide a large number of active hydroxyl groups that can be used in different adhesive systems, such as PU, epoxy, formaldehyde-based, and polymeric water-based adhesives [40]. Such liquefaction products are promising sources of bio-polyols due to their large availability of raw materials, rich in hydroxyl groups, and stable aromatic polymer structure [43,44].

Selection of appropriate raw materials and liquefaction conditions including solvent, catalyst, process temperature, and residence time, is however challenging because it does not only influence the liquefaction conversion degree, which is often examined by calculating liquefaction yield (LY) or residue content, but also affects the chemical and physical properties of the obtained bio-polyols, especially the hydroxyl numbers varying from 100 to 1043 mg·KOH/g [44,45]. The number of hydroxyl groups in polyols and the structure of both polyols and isocyanates are critical factors for preparing PU adhesives [46,47]. Zhou et al. [48] liquefied banana pseudo-stem in a solvent mixture of polyethylene glycol 400 (PEG 400) and glycerol catalyzed by sulfuric acid under the temperature from 130–170 °C for 90 min with a solvent-to-biomass ratio of 6. The authors reported that the liquefaction temperature, time, and catalyst amount significantly affected the LY. The obtained liquefied banana pseudo-stem (LBPP) had an increasing hydroxyl number from 294.8 to 370.2 mg·KOH/g by prolonging liquefaction time from 30 to 150 min. It was reported that the thermal stability of PU adhesives prepared from PEG 400 and 4,4′-diphenylmethane diisocyanate (4,4′-MDI) was improved; however, the adhesive bond strength was decreased by substitution of PEG 400 with LBPP. Bio-polyols from the liquefaction of alder wood at different conditions, i.e., solvent type, reaction temperature, and time, has a hydroxyl number value of 214–687 mg·KOH/g [27]. By studying the elasticity of the PU resins, they found that the PU resin with the NCO:OH ratio of 1:1 had a lower decomposition rate and higher storage modulus due to high cross-linking density compared to the resins with a ratio of 1.2:1.

Other parameters, such as isocyanate types, the molar ratio of isocyanate to hydroxyl groups, and catalyst can also affect the performance of PU adhesives. Lee and Lin [28] prepared PU adhesives by reacting liquefied wood (LW) from liquefaction of China fir (Cunninghamia lanceolata) and Taiwan Acacia (Acacia confusa) with three different isocyanates, i.e., pMDI, Desmodur L, and Desmodur N. The results showed that the liquefaction efficiency of China fir was higher than that of Taiwan Acacia due to the lower cellulose content in China fir. PU based on LW and Desmodur L had a better gel time than the PU based on LW and Desmodur N or pMDI. The gel time of PU adhesives was also affected by the NCO:(OH+COOH) ratio, surfactant, and catalyst. An increase of NCO:(OH+COOH) molar ratio from 1.0 to 2.0 led to higher dry and wet bonding strength for PU adhesives prepared both from Taiwan acacia and China fir with the same isocyanate. Lee and Chao [29] improved the tensile properties and thermal stability of thermoplastic polyurethane (TPU) adhesives prepared from polytetramethylene ether glycol (PTMG) and 1,6-hexamethylene diisocyanate (HDI) by adding liquefied cedar. TPU with added liquefied cedar (Cryptomeria japonica) had more hydrogen bonding as a result of higher tensile strength than TPU prepared with PTMG and HDI. Juhaida et al. [30] reported a lower bond strength of PU adhesives from liquefied kenaf core, toluene-2,4-diisocyanate (TDI), and 1,4-butanediol than of commercial PU adhesives. They claimed that the use of up to 1% catalyst could induce effective working time for the adhesive to be reactive on the wood surface. Daneshvar et al. [46] prepared PU adhesives from liquefied beech sawdust and pMDI or MDI with different NCO:OH molar ratios from 0.7 to 1.7. The authors reported that the highest lap shear strength was achieved at an NCO:OH ratio of 1.7 for both pMDI and MDI, which is related to excess free NCO groups; the lap shear strength of PU based on pMDI was higher than that of PU based on TDI. Sankar and Yan [31] blended pMDI and liquefied bark with castor oil or polyethylene glycol for preparing two-component PU adhesive. It was found that the formulations containing liquefied bark showed shorter gel time, better thermal stability, and comparable mechanical properties compared to those without liquefied bark, which was due to the catalytic function of the metal salts present in the liquefied bark.

There are still limited publications related to PU adhesives based on liquefied biomass, especially with the use of sawdust from Norway spruce (*Picea abies*). Normally, bio-polyols are obtained from the liquefaction products by removing solid residues. This paper intended to use crude polyol from an optimized liquefaction condition with a high LY for making PU adhesives. It is hypothesized that the solid residues in the crude would act as natural fillers in the PU adhesives and contribute to their performance. Comparison with PU adhesives prepared after purification of the crude polyol, i.e., by removing solid parts, would allow a knowledgeable decision whether such an energy and chemical demanding step is necessary. The impact of NCO:OH molar ratios on the chemical structure, mechanical properties, and thermal properties of the PU adhesives was investigated and discussed.

## 2. Materials and Methods

### 2.1. Materials

Norway spruce (*Picea abies* (L.) H. Karst) sawdust was collected from JG Andersson’s Söner AB (Linneryd, Sweden). The wood sawdust was milled to powders with a particle size of 1 mm using the grinding mill Polymix PX-MFC 90 D (Kinematica, AG, Luzern, Switzerland). Ethylene glycol (EG) produced by VWR International BVBA (Leuven, Belgium) was used as liquefaction solvent and sulfuric acid (95% purity) produced by VWR International S.A.S. (Fontenay-sous-Bois Cedex, France) was used as a liquefaction catalyst. 1,4-dioxane (VWR International S.A.S., Fontenay-sous-Bois Cedex, France) was used for the purification of liquefied wood. For PU preparation, chemicals used were pMDI (I-BOND^®^ PB PM 4350) as isocyanate kindly provided by Huntsman (Botlek-Rotterdam, Netherlands), dibutyltin dilaurate (95%) (Sigma-Aldrich Chemie GmbH, Deisenhofen, Germany) as reaction catalyst, and glycerol (Sigma-Aldrich Chemie GmbH, Deisenhofen, Germany) as a chain extender.

For determining the acid number of the polyols, pyridine, phenolphthalein, and sodium hydroxide were used and produced by Sigma-Aldrich Chemie GmbH (Deisenhofen, Germany). For measuring the hydroxyl group number, chemicals including pyridine, phthalic anhydride, imidazole, cresol red indicator, potassium hydroxide (KOH), and methanol were used and produced by Sigma-Aldrich Chemie GmbH (Deisenhofen, Germany).

### 2.2. Liquefaction Process

Milled wood sawdust was dried in the oven under 103 ± 2 °C for 24 h before liquefaction. For each liquefaction batch, 50 g oven-dried wood sawdust, 150 g EG, and 4.5 g sulfuric acid were charged to a 1 L three-neck glass reactor submerged in an oil bath and connected with a mechanical stirrer and a water condenser. The liquefaction was conducted under temperature between 150 and 190 °C for 90 min. The liquefaction was halted by removing the reactor from the oil bath and cooling to room temperature.

The liquefaction products were diluted in a mixture of 1,4-dioxane:water (4:1) and centrifuged (Heraeus Multifuge X1, Thermo Fisher Scientific, Osterode, Germany) at 1000 rpm for 10 min for removing solid contents. The 1,4-dioxane and water were then removed from the filtrates by rotary evaporation with IKA RV 8 (IKA, Staufen, Germany). The solid residues were dried in the oven at 103 °C for 24 h.

Liquefaction yield was calculated based on the equation below:

(1) $$Liquefaction\;yield\;\left(LY\right)\;\left(\%\right)=\left(1-\frac{M_{sr}}{M_{r}}\right)\times 100$$

where *M_sr_* is the oven-dried mass of solid residues after liquefaction and *M_r_* is the oven-dried mass of raw materials before liquefaction.

Two more liquefactions were performed with the same conditions with the highest LY. After the liquefaction process, one product without removing solid residues was directly used as crude LW (CLW) and another one, named as PLW, was purified by removing the solid residues using the dilution-centrifuge-evaporation method described above. These two polyols were used for further characterization and PU adhesive preparation.

### 2.3. Characterization of Polyols

CLW and PLW were characterized with respect to their acid and hydroxyl group number, viscosity, and pH value. The viscosity of polyol samples was analyzed by using a TQC DV1400 viscometer (Proinex, Capelle aan den IJssel, The Netherlands). The pH values of the polyols were measured with an IS 2100 L pHenomenal pH meter (VWR, Darmstadt, Germany).

The acid number of polyols was assessed according to the ASTM standard D4274-99 [49]; 0.5 g of polyol sample, 60 mL pyridine, 10 mL distilled water, and 0.5 mL of phenolphthalein solution were weighed into each of two 250 mL Erlenmeyer flasks and mixed by magnetic stirrers. The mixed solution was titrated with standard 0.1 N sodium hydroxide (NaOH) solution to a pink endpoint. Each sample was analyzed in triplicate and the average value is presented. The acid number in mg·KOH/g was calculated by following Equation (2).

(2) $$Acid\;number\;\left(\mathrm{mg}\cdot \mathrm{KOH}/g\right)=\frac{\left(V_{1}-V_{2}\right)\cdot N\cdot 56.11}{W}$$

where *V*_1_ (mL) is the amount of NaOH solution for titration of polyol sample, *V*_2_ (mL) is the amount of NaOH solution for titration of the blank sample, *N* (mol/L) is the concentration of NaOH solution, 56.11 (g/mol) is the molar mass of KOH, and *W* (g) is the polyol sample mass.

The hydroxyl number of polyols was also determined according to the ASTM standard D4274-99 [49]; 0.5–1 g of polyol sample was added into a three-neck round bottom flask and 25 mL of the esterification reagent (phthalic anhydride: imidazole: pyridine = 116 g: 18 g: 700 mL) was then added and mixed with the polyol sample until it was dissolved using a magnetic stirrer. The flask was kept in a water bath at 98 °C for 15 min. After cooling, 50 mL of pyridine and 10 mL distilled water were added and allowed to mix for an additional 2 min. Next, 0.5 mL of indicator solution (1% cresol red solution in pyridine) was added into the flask and the solution was titrated with 0.5 mL/L KOH solution in methanol. The equivalence point was determined by the change of color from pink to dark green. Samples in triplicate for each polyol were analyzed. The hydroxyl (OH) number was determined according to the following Equation (3)

(3) $$OH_{number}\left(\mathrm{mg}\cdot \mathrm{KOH}/g\right)=\frac{\left(V_{3}-V_{4}\right)\cdot N_{KOH}\cdot 56.11}{m}+{\mathrm{Acid}}_{\mathrm{number}}$$

where *V*_3_ (mL) is the amount of KOH solution for titration of polyol sample, *V*_4_ (mL) is the amount of KOH solution for titration of the blank sample, *N_KOH_* (mol/L) is the concentration of KOH solution, 56.11 (g/mol) is the molar mass of KOH, *m* (g) is the polyol sample mass, and *Acid_number_* (mg·KOH/g) is the acid number of the polyol sample.

### 2.4. Preparation of PU Adhesive

The isocyanate groups (NCO) in pMDI were determined as 31.5%, according to ASTM standard D 2572-97 [50]. The adhesive formulations were prepared in two steps. Initially, component A as a hydroxyl (OH) donor was made by mixing CLW or PLW with glycerol (2% based on the mass of polyol) and was stirred at 400 rpm for 3 min at room temperature. Component B was prepared by blending pMDI and DBTDL (0.05% catalyst based on the pMDI mass). Component B was then introduced to component A and the mixture was stirred at 400 rpm for 3 min at room temperature. The amount of polyol and pMDI was taken based on their respective NCO and OH groups at a NCO:OH molar ratio of 0.5:1, 1:1, 1.5:1, and 2:1. In total, eight PU adhesives were made, as shown in Table 2. The prepared PU adhesives were directly applied to wood samples. Some parts of the adhesives were cured in a climatized room (20 °C and 65% RH) for more than two weeks. The cured PU adhesives were then milled to powders of ≤ 1mm and used for chemical characterization and thermal analysis.

### 2.5. Bonding Strength of PU Adhesives

The bonding strength of PU adhesives was determined according to the European standard EN302-1: 2013 [51]. PU adhesives were applied to European beech (*Fagus sylvatica* L.) wood (conditioned previously at 20 °C and 65% RH for two weeks) measuring 130 × 300 × 5 mm^3^ with a thin bondline of approximately 0.1 mm. The application amount of the adhesives on the wood surface was 200 g/m^2^. The glued assemblies were then pressed at 1.5 MPa at room temperature for 24 h. The assemblies were conditioned at 20 °C and 65% RH for 7 days and then converted to stripes with an overlapped joint area of 20 × 10 mm^2^ in the middle. The longitudinal tensile shear strength of wood assemblies was evaluated by lap shear tensile strength using a universal testing machine (MTS Exceed E43, MTS Systems Corporation, MN, USA) of 10kN capacity with a constant loading rate of 2 mm·min^−1^. Ten specimens were tested for each type of PU adhesive.

### 2.6. Adhesive Penetration

The adhesive penetration on the cross section of wood assemblies after finishing with a sledge microtome (WSL lab microtome, Birmensdorf, Switzerland) was observed under a motorized Olympus BX63F light microscope (Olympus, Tokyo, Japan) equipped with a DP73 color CCD cooled camera (max. 17.28 megapixel) and a cellSens Dimension 1.18 software (all from Olympus, Tokyo, Japan). For each adhesive type, 30 random areas with dimensions of 1100 × 600 (tangential × radial) µm^2^ were selected for measuring the maximum penetration (MP) of the PU adhesives according to previous studies [52,53]. The average distance of penetration of the five deepest adhesive objects from the bondline within each measuring region is calculated using Equation (4):

(4) $$MP=\frac{\sum_{1}^{5}\left(y_{i}+r_{i}-y_{0}\right)}{5}$$

where *MP* is the maximum depth of penetration (µm), *y_i_* is the centroid of adhesive object *i* (µm), *r_i_* is the mean radius of adhesive object *i* (µm), and *y*_0_ is the reference y-coordinate of the bondline interface (µm).

### 2.7. Analytical Characterization of Polyols and Cured PU Adhesives

The chemical structure of cured PU adhesives, raw wood sawdust, and liquefied polyols were analyzed by a Frontier FTIR (Flourier Transform Infrared) spectrophotometer (PerkinElme, Waltham, MA, USA). The analysis was performed averaging 32 scans with a resolution of 4 cm^−1^ in a wavelength range between 4000 and 650 cm^−1^.

The thermal stability of polyols and cured PU adhesives were evaluated by a TG 209 F1 IRIS equipment (Netzsch-Gruppe, Selb, Germany) with a temperature range of 30 to 800 °C at a heating rate of 10 °C·min^−1^ under nitrogen flow.

### 2.8. Statistical Analysis

One-way ANOVA and Tukey’s significant difference test were performed using a SPSS version 25.0 statistical software package (IBM Corp., Armonk, NY, USA) on the tensile shear strength and adhesive penetration results for the analysis of variance at a 95% confidence interval (*p* < 0.05).

## 3. Results and Discussion

### 3.1. Liquefaction Yield and Polyol Characteristics

**Figure 1:**
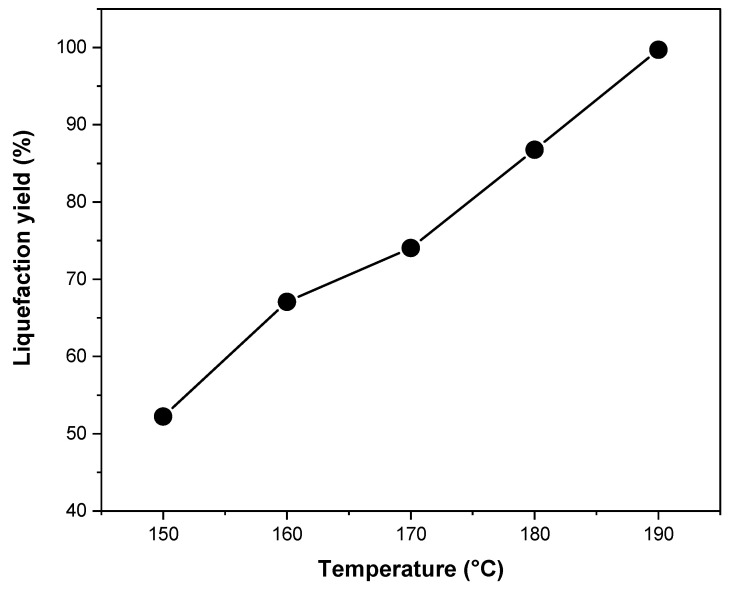
Effect of temperature on the liquefaction yield. Liquefaction time, 90 min; sulfuric acid, 3%; liquefying solvent, EG; solvent to biomass ratio, 3:1.

The liquefaction yield (LY) results of spruce wood sawdust in EG solvent (solvent to biomass ratio of 3:1) with 3% sulfuric acid catalyst (based on the solvent mass) for 90 min at different liquefaction temperatures are presented in Figure 1. LY was improved considerably by increasing the temperature from 150 to 190 °C. The highest LY was 99.7% at 190 °C, which indicates that almost the entire sawdust raw materials were liquefied. The increased temperature could accelerate liquefaction reaction because of the lower surface tension of the solvents, accelerated permeation of the catalyst, and promoted diffusion of decomposed components of wood into the solvent [54]. A rapid liquefaction takes place in the first 15–30 min for the degradation of more accessible biomass components like lignin, hemicelluloses, and amorphous cellulose, whereas the liquefaction proceeds at a slower rate after 30 min for the degradation of the less solvent-accessible crystalline cellulose [44]. Thus, high temperature and prolonged liquefaction time, i.e., 90 min in this study, ensured a high liquefaction efficiency. LY is also influenced by many factors, such as biomass type, solvent and catalyst types, biomass to solvent ratio, catalyst concentration, temperature, and reaction time. It is generally accepted that the liquefaction of wood at a solvent to biomass ratio of 3:1 is most effective, while a lower ratio than 3:1 will lead to polyols with an extremely high viscosity [44,55].

CLW prepared with the highest LY of 99.7% and its purified derivative PLW were analyzed for their OH and acid numbers, viscosity, and pH values (Table 3). The OH number of CLW of 825 ± 11 mg·KOH/g was considerably higher than that of PLW (623 ± 8 mg·KOH/g). As reported by others, the number of hydroxyl groups in liquefaction polyols varies among different biomass type, and it is, for example, 200–1043 mg·KOH/g for wood, 109–430 mg·KOH/g for agricultural cops, and 132–585 mg·KOH/g for bark [31,44,45,56]. The high OH number of CLW was attributed to the cleavage of the ether linkage of lignin and cellulose units, and the decrease in the OH number of PLW could be due to the dehydration and evaporation of alcohols from CLW [29,57]. Acidic substances normally exist in wood components and are increased during the liquefaction due to the oxidation of polysaccharides or depolymerization of the wood polymers [41]. The acid number of CLW of 48.2 ± 1.1 mg·KOH/g was slightly higher than the reported range of 12–41 mg·KOH/g from the literature [44,58], but it was not significantly changed after purification. This indicated that no further oxidation occurred during the purification. The viscosity and pH values of CLW and PLW were almost identical and agreed with the literature, i.e., viscosity in the range of 300–7880 mPa·s [44,59,60] and a pH of −0.50–1.63 [61,62].

**Figure 2:**
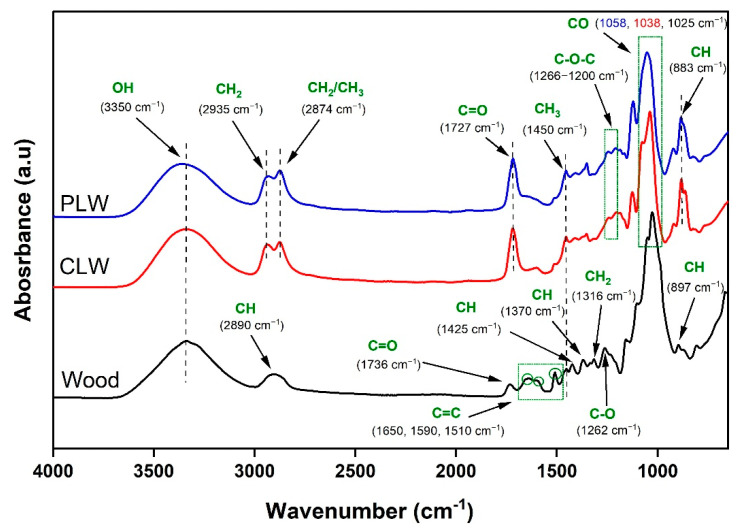
Fourier transform infrared spectroscopy (FTIR) spectra of spruce wood sawdust, crude liquefied wood (CLW) and purified liquefied wood (PLW).

The FTIR spectra of spruce wood sawdust and the derived bio-polyols after liquefaction are presented in Figure 2. Wood, as a natural polymeric material, is composed of carbonyl, hydroxyl, ester, and ethyl linkages along with carbon–hydrogen bonds [63]. The stretching bands at 3350 cm^−1^ and 2890 cm^−1^ were respectively associated with the OH and CH bonds in cellulose, hemicelluloses, and lignin. A vibration at 1736 cm^−1^ corresponded to C=O stretching in unconjugated ketones, aldehydes, and carboxyl in xylan and hemicelluloses [63,64]. The broad band between 1650 cm^−1^ and 1590 cm^−1^, and the peak at 1510 cm^−1^, represented the C=C stretching of the aromatic ring in lignin [65]. Lignin bands were also found at 1450 cm^−1^ for asymmetric CH_3_ bending in methoxyl groups in lignin structure, 1425 cm^−1^ for aromatic skeletal vibration with in-plane CH deformation, and 1262 cm^−1^ resulting from syringyl ring breathing and C-O stretching in lignin and hemicelluloses [64,65,66]. The peaks specifically assigned to cellulose were at 1370 cm^−1^ for CH deformation, 1316 cm^−1^ for CH_2_ bending, 1025 cm^−1^ for C-O stretching, and 897 cm^−1^ for symmetric CH stretching vibration [67,68,69]. The chemical structure of CLW and PLW were similar but apparently different from that of raw wood sawdust. The absorbance band at 3350 cm^−1^ corresponding to OH groups were observed in both polyols. Two intensive peaks were observed at 2935 cm^−1^ and 2874 cm^−1^, which corresponded to CH_2_ and CH_3_ stretching. These, together with the disappeared peaks at 1650 cm^−1^, 1590 cm^−1^, and 1510 cm^−1^ in the spectra of CLW and PLW, compared to raw wood, confirmed the fragmentation of the chemical components of wood [70,71]. The carbonyl peak at 1736 cm^−1^ in the wood sawdust was shifted to 1727 cm^−1^ in CLW and PLW, which was attributed to the hydrolysis of cellulose and possible formation of levulinic acid and carboxylic acid, and also to the characteristics of aldehydes, ketones, and ester groups [72,73,74]. A bending vibration between 1266 and 1200 cm^−1^ was characteristic of the C-O-C groups of the ether bonds that originated from lignin [75,76]. The C-O bond stretching vibration was shifted from 1025 cm^−1^ in wood sawdust to 1038 cm^−1^ in CLW and to 1058 cm^−1^ in PLW. This is mainly associated with the cleavage of the glucoside bonds in the cellulose and the formation of new alcohol-glycoside [44]. The FTIR spectra confirmed that the chemical structure of wood sawdust was changed considerably under the liquefaction process and the obtained polyols contained a great number of hydroxyl groups appropriate for producing PU. In addition, the purification process did not cause changes to the chemical structure of bio-polyol.

**Figure 3:**
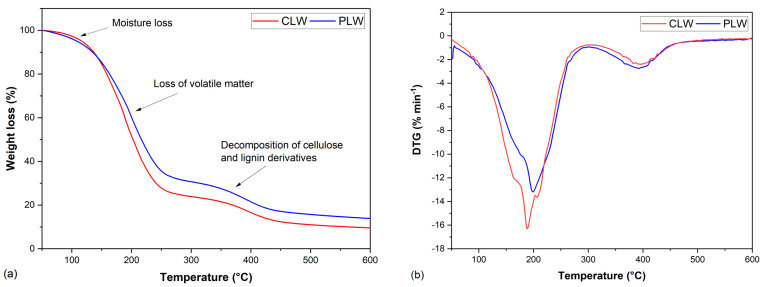
(**a**) Mass loss of bio−polyols; (**b**) Derivative weight of bio−polyols.

The thermal degradation behavior of CLW and PLW was studied by thermogravimetric (TG) and derivative thermogravimetric (DTG) analysis (Figure 3a,b). The weight changes of CLW and PLW at elevated temperatures are shown in Figure 3a,b illustrates the slope of the TG curve, which indicates the decomposition temperatures of the polymers. Both CLW and PLW went through two different stages. The first stage occurred between 100 and 300 °C due to the evaporation of water and low-molecular-weight compounds, and the defragmentation and degradation of the liquefied products [77]. The second stage took place at around 300–450 °C, where the liquefied products introduced by lignin and cellulose underwent further thermal decomposition [77]. Two main degradation peaks were observed for CLW and PLW, as shown in Table 4. The first temperature at which maximum weight loss (T_max1_) occurred was 189 °C for CLW and 200 °C for PLW, and the second maximum decomposition temperature (T_max2_) at 396 °C was for CLW and 391°C for PLW. The total weight loss of CLW was approximately 4.5% more than that of PLW, indicating a removal of water and alcohol during the purification process.

### 3.2. Performance of PU Adhesives

**Figure 4:**
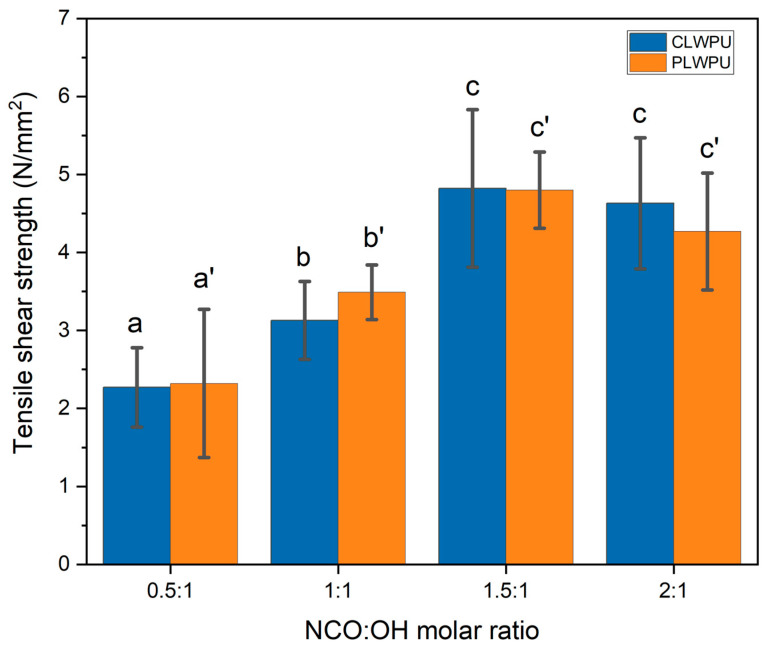
Tensile shear strength of wood samples glued with PU adhesives prepared from CLW or PLW polyols. The statistical differences between mean values at a 95% confidence interval (*p* < 0.05) are indicated by different letters and were assessed by using one-way ANOVA and Tukey’s significant difference test.

The tensile shear strength of wood samples bonded with CLWPU and PLWPU adhesives at various NCO:OH molar ratios are shown in Figure 4. The bonding strength of assemblies was improved by increasing the NCO:OH molar ratio from 0.5:1 to 1.5:1. These improvements were found to be statistically significant (*p* < 0.05). The higher mean value of tensile shear strength was 4.82 ± 1.01 N/mm^2^ for CLWPU and 4.80 ± 0.49 N/mm^2^ for PLWPU, obtained at the NCO:OH ratio of 1.5:1. This could be attributed to the formation of a high crosslinking density in the formulation with a higher NCO:OH molar ratio up to a certain level. At a low NCO:OH ratio, the quantity of NCO groups was insufficient to react with the OH groups of polyols for forming a strong urethane network [46], and thus relatively poor bonding strengths were obtained for PU formulations with NCO:OH ratios of 0.5:1 and 1:1. The bonding strength slightly decreased in the formulations with NCO:OH ratio of 2:1 for both CLWPU and PLWPU, although the changes were not statistically significant. The excess NCO groups in the formulations may cause side-reactions, i.e., reactions of NCO with the urethane groups to form allophanates or reactions of NCO with amine (NH_2_) groups to form a biuret structure [28,47,58]. These various side-reactions could possibly result in a wide distribution of cross-linking density within the adhesive network, and thus a slight reduction in the bonding strength of the PU adhesives prepared with a NCO:OH molar ratio of 2:1 compared to the ones of 1.5:1. It should be noted that the bonding strength of CLWPU and PLWPU at the same NCO:OH molar ratio were identical, although CLW showed a considerably higher OH number than PLW. This might be due to the lower reactivity of OH groups in CLW polyol as compared with PLW polyol [27].

**Figure 5:**
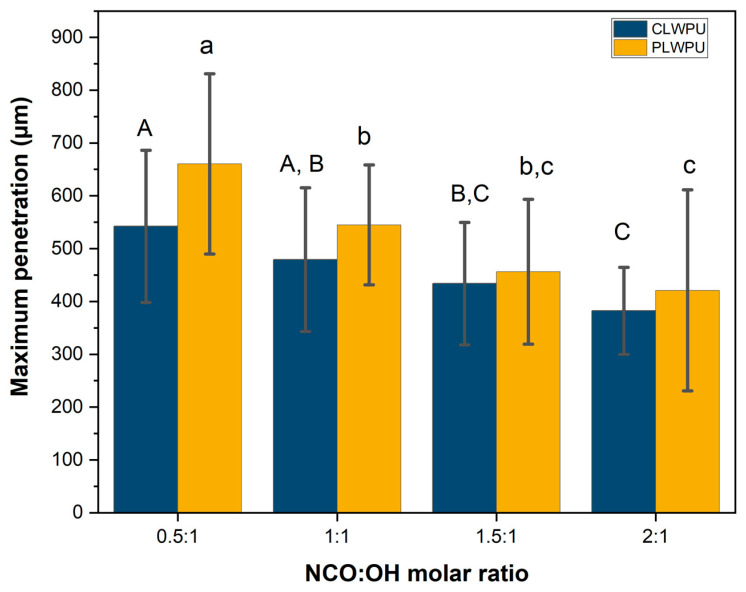
Maximum penetration of CLWPU and PLWPU adhesives in beech wood assemblies. The statistical differences between mean values at a 95% confidence interval (*p* < 0.05) are indicated by different letters and were assessed by using one-way ANOVA and Tukey’s significant difference test.

**Figure 6:**
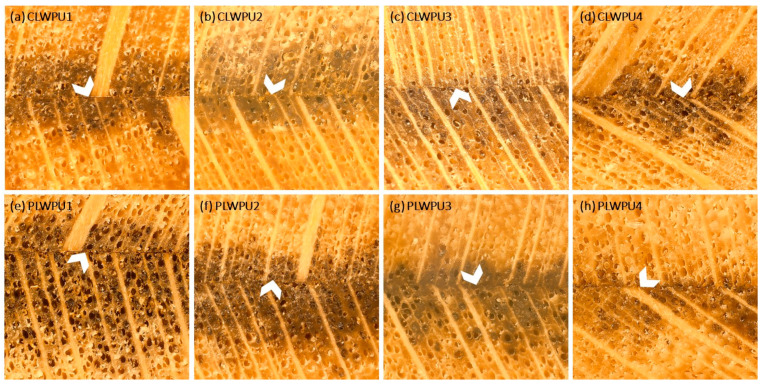
Light microscopy images of adhesive penetration: (**a**–**d**) LWPU1-4 was made with a NCO/OH ratio from 0.5:1 to 2:1; (**e**–**h**) PLWPU1-4 was made with a NCO/OH ratio from 0.5:1 to 2:1. Arrow heads are showing the bondlines.

The maximum penetration (MP) values of the CLWPU and PLWPU adhesives were changed considerably at various NCO:OH molar ratios (Figure 5). MP decreased by increasing the NCO:OH ratio from 0.5:1 to 2:1 for both CLWPU and PLWPU. PLWPU formulations showed generally a better penetration than the CLWPU ones, which could be attributed to the lower viscosity of the PLW polyol. The adhesive penetration of both CLWPU and PLWPU were dominated by the flow through vessels and fibers, and appeared in partial filling of the lumen due to the wetting of the lumen surface by a thin film of resin (Figure 6). There was no visible bondline (layer of adhesives between two adherends) in CLWPU 1 and PLWPU1 with the highest penetration because most parts of the PU containing unreacted polyols penetrated into the wood. This could also be related to the lowest tensile shear strength which occurred in CLWPU1 and PLWPU1. Although the bonding strength of CLWPU and PLWPU had a significant increment by increasing the NCO:OH up to 1.5:1, finding a meaningful correlation between the strength improvement and adhesion penetration is rather challenging [78,79].

### 3.3. Analytical Characterization of Cured PU Adhesives

#### 3.3.1. FTIR Spectra

**Figure 7:**
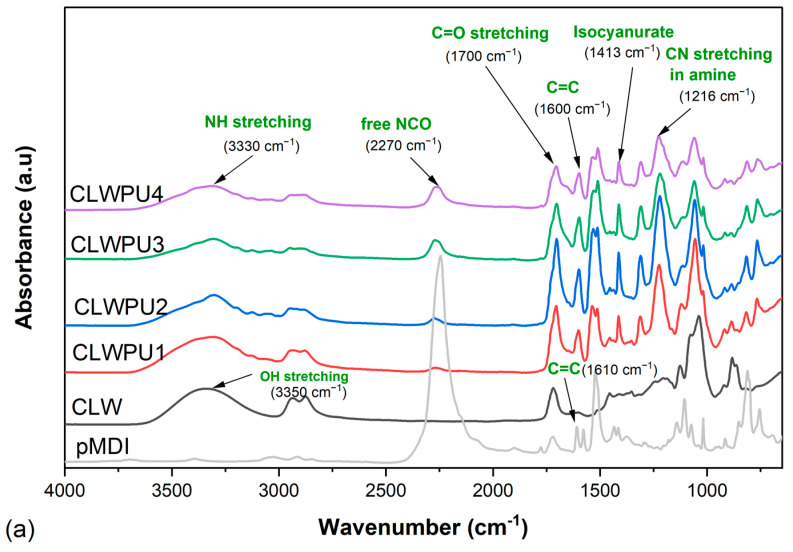

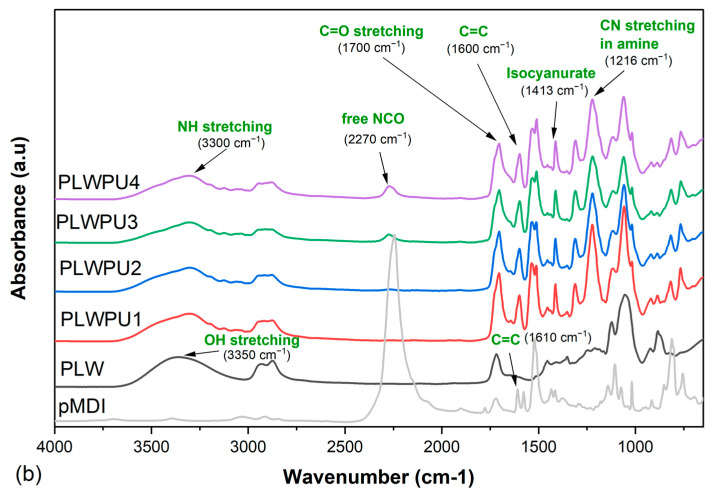
FTIR spectra of cured CLWPU1-4 with a NCO:OH molar ratio from 0.5:1 to 2:1 (**a**) and of cured PLWPU1-4 with a NCO:OH molar ratio from 0.5:1 to 2:1 (**b**).

Figure 7 shows the FTIR spectra for the cured CLWPU and PLWPU adhesives at various NCO:OH molar ratios. The absorption band at 3330 cm^−1^, corresponding to NH stretching and the peak at 1700 cm^−1^ attributed to the C=O stretching, indicated the reaction between bio-polyols and pMDI to form urethane linkages [30,80]. The peak at 2270 cm^−1^ was associated with free NCO groups at the end of the PU polymer chain and in the neat pMDI. The absorption of this peak decreased obviously in the cured adhesives compared to neat pMDI, which indicates that most of the free NCO groups were consumed. However, this peak appeared with a higher intensity in PU, with a higher NCO:OH molar ratio for both CLWPU and PLWPU. The adhesive formulations with CLW polyol showed a greater free NCO band vibration than the ones with PLW. This interesting finding proved the previous assumption of higher reactivity of the OH groups in the PLW than the CLW, which means that the reactivity of OH groups was improved by the purification procedure. This might be due to the removal of secondary OH groups with a relatively low reactivity from CLW [27,81]. The peaks observed at 1600 cm^−1^ for CLWPU and PLWPU, and the peak at 1610 cm^−1^ for neat pMDI, were associated with the conjugated double bonds in the aromatic ring of the hard segment originated from pMDI [46]. In addition to the reaction between polyols and isocyanates, two further reactions may also have occurred: (a) reaction between pMDI and water molecules from the atmosphere to form amine groups by releasing CO_2_ gas, which was attributed to the peak of 1216 cm^−1^, and (b) reaction within the isocyanate groups to form isocyanurate, i.e., the peak at 1413 cm^−1^ [6,82,83]. The formation of amine groups decreased by increasing the NCO:OH molar ratios. This was noticeable even during the adhesive preparation by the considerable release of CO_2_ gas at a lower NCO:OH molar ratio.

#### 3.3.2. TGA

**Figure 8:**
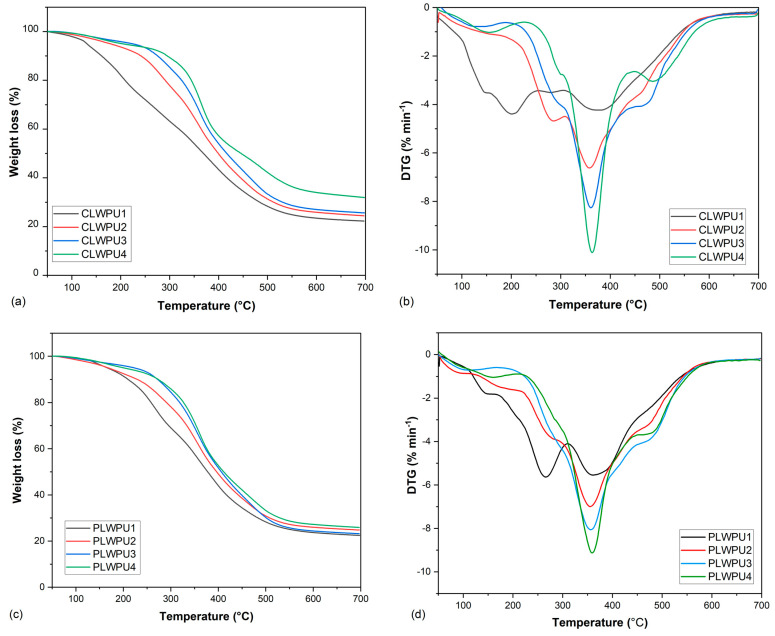
Mass loss of CLWPU1−4 (**a**) and PLWPU1−4 (**c**); first derivative of CLWPU1−4 (**b**) and PLWPU1−4 (**d**).

Figure 8 shows the thermal degradation behavior of the cured CLWPU and PLWPU adhesives at various NCO:OH molar ratios. There were considerable differences in the degradation patterns by increasing the NCO:OH molar ratio from 0.5:1 to 2:1. The initial weight loss was due to the evaporation of water and the release of volatile matters [46]. The initial degradation temperatures (T_onset_ in Table 5) were shifted respectively from 111 to 227 °C and from 110 to 215 °C for CLWPU and PLWPU, by increasing the NCO:OH molar ratio from 0.5:1 to 2:1. This shift can be due to the increasing hard segment content in the PU. The T_max1_ for CLWPU and PLWPU at the NCO:OH molar ratio of 0.5:1 were 202 and 265 °C, respectively. These peaks have may been caused by the cleavage of urethane bonds, which led to the formation of CO_2_, alcohols, amines, aldehydes, and CO [27,84]. With an increasing molar ratio in both CLWPU and PLWPU adhesive formulations, the T_max1_ was shifted to higher temperatures and then disappeared. The T_max2_ occurred at a temperature range of 355–380 °C for all PU formulations, which corresponded to about 30–52% for CLWPU and 34–45% for PLWPU formulations. This might be due to the intensified decomposition of urethane bonds and decomposition of soft segments, i.e., polyols [85]. The formulations with high NCO:OH molar ratios showed T_max3_ at a temperature over 450 °C, which referred to the degradation of hard segments, such as pMDI and aromatic lignin moieties [86]. The thermal stability of cured PU adhesives was improved considerably by increasing the NCO:OH molar ratio in both CLW- or PLW-based formulations. This might be due to excess free isocyanate (pMDI), which formed a higher crosslinking density [87].

## 4. Conclusions and Outlook for Future Work

In this paper, two bio-polyols were prepared by the liquefaction of spruce wood sawdust and were used for the preparation of PU adhesives by reacting with polymeric 4,4′-diphenylmethane diisocyanate (pMDI). A high liquefaction yield of 99.7% was achieved at 190 °C for 90 min in presence of sulfuric acid with a concentration of 3% and ethylene glycol with a solvent-to-wood ratio of 3:1. The chemical characteristics and thermal behavior of the crude (CLW) and purified (PLW) liquefied product were analyzed. The results showed that the purification process changed the hydroxyl group content and viscosity of the polyols due to the removal of secondary OH groups, which also led to a higher reactivity of PLW than CLW. The PU adhesives prepared from CLW or PLW with pMDI at various NCO:OH molar ratios presented different chemical structures, adhesion strengths, and thermal behavior. FTIR spectra clearly indicated the reaction between polyols and pMDI to form urethane bonds together with some other side reactions. The tensile shear strength of CLWPU and PLWPU adhesives increased by increasing the NCO:OH molar ratios up to a ratio of 1.5:1, at which the highest shear strength was achieved. The thermal properties of the cured adhesives improved considerably by increasing the NCO:OH molar ratios. However, CLWPU and PLWPU adhesives at the same NCO:OH molar ratios turned out to have similar chemical structures, adhesion strengths and thermal degradation behaviors.

This study supports the hypothesis that bio-polyols from biomass liquefaction are suitable for producing PU adhesives. The crude liquefaction products without purification are appropriate for such a purpose. Both CLW and PLW polyols contain a great number of hydroxyl groups. Due to the complex chemical composition of the polyols from liquefaction, further investigation into the polyol chemical structure will facilitate the selection of certain types of isocyanates. The specific parameters for synthesis of PU should also be researched for producing adhesives with desired properties.

## Figures and Tables

**Table 1:** Development of bio-based polyurethane adhesives.

Year	Developing Strategies	Remarks	Reference
1991	Poly(chloroprene)-castor oil based polyurethane adhesives	Good adhesion for rubber to rubber bonding	[10]
2001–2021	Non-isocyanate polyurethane adhesives	Need of high curing temperature; renewability; non-toxicity	[11,12,13,14,15,16]
2003–2021	Castor oil based polyurethane adhesives for wood bonding	Highly crosslinked polymeric structure; low cost; renewablility	[17,18,19,20,21,22,23,24]
2003	Polyurethane adhesives based on polyester polyols from potato starch and natural oils	Good bonding property; highly cross-linked structure; good water resistance	[25]
2005	Polyurethane wood adhesives based on palm kernel oil	Good adhesion property; low cost	[26]
2008–2020	Polyurethane adhesives based on bio-polyols from liquefaction of lignocellulosic materials	Abundant availability of raw materials; rich in hydroxyl groups; stable aromatic polymer structure	[27,28,29,30,31]
2011	Polyurethane wood adhesives based on canola oil	Low cost; good bonding property and chemical resistance; superior hot water resistance	[32]
2016	Polyurethane adhesive based on kraft lignin as a polyol	High reactivity of the hydroxyl groups in kraft lignin; use of industrial waste	[33]
2017	Moisture curable silane-terminated polyurethane adhesives	Avoidance of CO_2_ production	[34,35,36]
2017	Polyurethane wood adhesives from crude glycerol-based polyols	Use of the waste stream of biodiesel production; low cost; renewablility	[37]
2018	Application of oxazolidine compounds in one-component polyurethane adhesives	Decreased bubble number and size; better bondability	[38]
2020	Polyurethane wood adhesives based on polyester polyol from soybean oil	Improved performance by addition of additives	[39]
2021	Solvent-free polyurethane adhesives based on castor oil and organic diisocyanates	Fast curing; low adhesion	[13]

**Table 2:** PU adhesives made from CLW and PLW polyols and their corresponding NCO:OH molar ratio.

NCO:OH Molar Ratio	CLWPU	PLWPU
0.5:1	CLWPU1	PLWPU1
1:1	CLWPU2	PLWPU2
1.5:1	CLWPU3	PLWPU3
2:1	CLWPU4	PLWPU4

**Table 3:** Hydroxyl and acid numbers, viscosity, and pH of crude (CLW) and purified liquefied wood (PLW).

Properties	CLW	PLW
OH number (mg KOH/g)	825 ± 11	623 ± 8
Acid number (mg·KOH/g)	48.2 ± 1.1	47.8 ± 1.4
Viscosity (mPa·s at 20 °C)	3900	3700
pH	0.19	0.22

**Table 4:** Thermal degradation properties of bio−polyols.

Material	T_onset_ (°C)	T_5wt.%_ (°C)	T_10wt.%_ (°C)	T_max1_ (°C)	T_max2_ (°C)	T_offset_ (°C)
CLW	50	117	137	189	396	489
PLW	50	109	134	200	391	499

**Table 5:** Thermal degradation stability of cured CLWPU and PLWPU adhesives.

Material	T_onset_ (°C)	T_5wt.%_ (°C)	T_10wt.%_ (°C)	T_max1_ (°C)	T_max2_ (°C)	T_max3_ (°C)	T_offset_ (°C)
CLWPU1	111	131	161	202	380	-	637
CLWPU2	100	179	241	285	358	-	614
CLWPU3	190	203	275	-	361	460	630
CLWPU4	227	224	295	-	363	487	630
PLWPU1	110	165	178	265	360	-	615
PLWPU2	114	169	230	-	355	-	608
PLWPU3	167	202	271	-	356	-	617
PLWPU4	215	224	274	-	359	460	618

## Data Availability

The data presented in this study are available on request from the corresponding author.
